# Towards the Exploration and Evolution of Insulin-like Venoms in Actiniaria (*Sea anemones*)

**DOI:** 10.3390/md22030136

**Published:** 2024-03-20

**Authors:** Alonso Delgado, Kyle S. Sozanski, Marymegan Daly

**Affiliations:** Department of Evolution, Ecology and Organismal Biology, The Ohio State University, Columbus, OH 43210, USA; sozanski.1@osu.edu (K.S.S.); daly.66@osu.edu (M.D.)

**Keywords:** insulin-like venom, venom, toxins, venomics, transcriptome, *Sea anemone*, selection, Actinaria, Cnidaria

## Abstract

Recent studies have elucidated the diversity of genes encoding venom in *Sea anemones*. However, most of those genes are yet to be explored in an evolutionary context. Insulin is a common peptide across metazoans and has been coopted into a predatory venom in many venomous lineages. In this study, we focus on the diversity of insulin-derived venoms in *Sea anemones* and on elucidating their evolutionary history. We sourced data for 34 species of *Sea anemones* and found sequences belonging to two venom families which have Insulin PFAM annotations. Our findings show that both families have undergone duplication events. Members of each of the independently evolving clades have consistent predicted protein structures and distinct dN/dS values. Our work also shows that sequences allied with VP302 are part of a multidomain venom contig and have experienced a secondary gain into the venom system of cuticulate *Sea anemones*.

## 1. Introduction

Venom is a biological toolkit comprised of cocktails of molecules that disrupt the physiology of an organism when it is injected (envenomated) by the organism that produced it. The delivery mechanisms, behaviors, and ecological interactions of envenomation can be studied across the animal tree of life because venom has convergently evolved across most major lineages [1]. Among known venomous organisms, Cnidaria is the oldest lineage in which venom has evolved. Cnidarians are unique among venomous organisms because they have a microscopic and decentralized venom delivery apparatus, the nematocyst. Nematocysts are made by a kind of cell unique to cnidarians (nematocytes) and are highly diverse in shape, size, and in spatial distribution across the body [2].

Among cnidarians, venom is best understood in the cnidarian subphylum Anthozoa (corals and *Sea anemones*), and particularly within the anthozoan order Actiniaria (*Sea anemones*). Venom is integral to the biology of *Sea anemones* because they use venom for a variety of functions, including prey capture, defense, digestion, and intraspecific competition [3,4]. Due to this biological breadth, the *Sea anemone* venom arsenal is highly diverse in its molecular constituents, with most species making an array of different compounds [5,6,7,8,9]. The compounds found in *Sea anemone* venom can be broadly categorized into larger functional categories, and include auxiliary toxins, allergens and innate immunity toxins, hemostatic and hemorrhagic activity toxins, mixed function enzymes, voltage-gated channel toxins, pore-forming toxins (cytolysins), protease inhibitors, and actiniarian toxins of unknown function [4,9,10,11]. At a genomic level, the genes encoding venom in any given species can vary in identity and in abundance, with abundance reflecting multiple copies of genes [12] or differential transcription (variation in normalized expression) [13] or both.

Studies of genes encoding venom in *Sea anemones* have shown that genes and gene families evolve under purifying selection [14,15]. This is distinct from genes and gene families for venom in other lineages, where diversifying selection predominates [16,17,18]. This initial assessment of evolutionary trends in the genes for venom in *Sea anemones* was based on a limited number of species and only members of the superfamily Actinioidea. Actinioidea is estimated to have diverged from other lineages of *Sea anemone* approximately 450 mya [19], making it significantly older than venomous lineages like snakes and cone snails, which are estimated to be <100 mya [16,17,18]. More recently, the taxonomic scope of study of evolution in venom genes of *Sea anemones* has expanded, but the functional breadth of the venom families considered remains limited, emphasizing neurotoxins [12,20,21,22].

For *Sea anemones*, as for many groups, the biomedical potential of venom has driven their study. This likely has played a role in the emphasis on neurotoxins, whose analogs in snakes and cone snails have strong potential for pharmaceutical application [23]. The peptide SHK1, originally isolated from the actinioidean *Stichodactyla helianthus*, has been explored for its potential in autoimmune disorders (reviewed in [22,24]) and its therapeutic potential has led to studies of its structure [25], function [24], and diversity [6,12,22]. Other *Sea anemone* venom constituents, like the amylase inhibitors helianthase [26] and magnificamide [27], represent novel biological solutions to common metabolic problems like the breakdown of carbohydrates [28]. Yet other molecules within *Sea anemone* venoms, like NV1 [22], have both genetic and structural similarities to human peptides and proteins and thus may offer biomedical insights.

Insulin is a widely occurring and well-studied peptide in vertebrate systems [29,30]. It is also found in non-vertebrates, although only a handful of studies have examined the function of insulins across the tree of life. In amniotes, insulin is produced by the pancreas and functions to regulate blood sugar levels by promoting glucose uptake and the absorption of glucose from the blood into liver, fat, and skeletal muscle cells. In non-vertebrates, insulin-like molecules are referred to as Insulin-Like Peptides (ILPs) and typically hold distinct functions. In insects, ILPs regulate metabolism and growth [31,32] and are a key factor in reproduction and molt signaling [33]. In nematodes, ILPs act as pathway antagonists, stopping L1 arrest during development [34]. In mollusks, ILPs have been tied to neural regulation [35]. In a few lineages, including bees [36], monotreme mammals [37,38], snakes [39,40], lizards [41], and cone snails [42,43], insulin has been recruited into the venom cocktail, where it may block site-specific channels and/or act indirectly on prey by disrupting insulin homeostasis. The best-studied ILP in a venomous system is in marine cone snails [17,42,43,44], in which the ILP acts antagonistically towards prey by causing hypoglycemic shock, enabling the snail to slowly eat their prey [44].

The transcriptome of *Sea anemones* contain transcripts that closely resemble the well-known venomous peptide conoinsulin from cone snails [45,46]. In a study that examined these peptides in the undescribed actinioidean *Sea anemone*, Oulactis sp., Mitchell and colleagues showed that the anemone-derived peptides showed signs of binding to both Kv1-3 binding sites and insulin growth receptors [45]. Similar sequences also occur in the genome of Nematostella vectensis [47]. Following the convention established for the cone snail peptides, we refer to the transcripts from cnidarians with high affinity to conoinsulins as cnidoinsulins.

*Sea anemone* cnidoinsulins are transcribed as a peptide with three chains (B + C + A) that undergoes post transcriptional modifications to cleave off the C chain and form a mature pro-peptide (B + A) [35]. The structure of the peptide offers an opportunity to examine selection across its functional chains. Work by Gibbs [16], Jouiaei [15], and their colleagues point to the possibility of certain regions in venom-encoding genes evolving rapidly under the influence of adaptive selection.

The only other known instance of an insulin-like venom is the protein VP302, originally discovered in the transcriptome of a swimming scorpion [48]. VP302 has also been identified in other cnidarians, including in transcriptomes of four species of Cerianthiaria [49], in the genome of *Hydra vulgaris* [50], and in clownfish-hosting *Sea anemones* [9]. Studies which annotate venom coding genes functional families have found that VP302 matches to the protein family (PFAM) insulin receptor growth factor [9,49].

The diversity and evolutionary history of venoms derived from insulins is poorly characterized for actiniarians [46]. The commonness of cnidoinsulin transcripts in species that span the *Sea anemone* phylogeny suggests that insulin-like venoms may be found across all *Sea anemones*, but their diversity is undocumented, both within taxa and across the breadth of Actiniaria. Fu and colleagues [46] interpret *Sea anemone* cnidoinsulins to have a single origin relative to other metazoans, but their study did not include sequences from non-anemone cnidarians or exemplars of VP302. The breadth of occurrence of VP302 and its evolutionary history within *Sea anemones* or Cnidaria is unknown.

Previous work on venom genes in actiniarians predict that insulin-like venoms will evolve under negative selection [14,15], but the appropriateness of generalization from neurotoxins to insulin-like venoms remains untested. Furthermore, genes encoding venom may undergo episodic and site-specific selection [14,15], and punctuated equilibrium may represent a more appropriate model in at least some actiniarian neurotoxins [12,14].

In this study, we document the diversity of insulin-like venoms in the transcriptomes of 34 species of *Sea anemone*. Additionally, we model the structure of exemplar insulin-like venoms based on sequences from each of the major clades of actiniarians from which we document insulin-like venoms. We analyzed the insulin-like venoms we recovered to infer (1) the evolutionary history and diversity of each type of insulin-like venom within Actiniaria and (2) the mode of selection for the genes encoding insulin-like venoms in *Sea anemones*. We explicitly consider both kinds of insulin-like venoms previously identified in *Sea anemones*, cnidoinsulins and VP302.

## 2. Results

### 2.1. Identification

We recovered transcripts identifiable as insulin-like venoms in 28 of the 34 species we studied. We found no sequences that matched insulin-like venoms in the transcriptome of the sole representative of superfamily Actinostoloidea, *Stomphia coccinea* (family Actinostolididae). We also found no insulin-like venoms in transcriptomes from the actinioideans *Epiactis prolifera* and *Macrodactyla doreensis* (both family Actiniidae), nor in those of the metridioidean *Lebrunia danae* (family Aliciidae).

The insulin-like venoms we recovered from across Actiniaria matched two previously described venom types: (1) cnidoinsulins, which matched to conoinsulins found in cone snails [51,52]; and (2) Venom Protein 302 (VP302) originally described in *Lychas mucronatus* (Chinese swimming scorpion). Transcripts identified as cnidoinsulins were recovered in 20 species. Transcripts with a high match to VP302 were recovered in 21 species. Nineteen species had both cnidoinsulins and VP302, and five species had no transcripts matching with either cnidoinsulins or VP302 (Table 1). We recover multiple isoforms for hit to both cnidoinsulin and VP302, which our phylogenetic analyses designate as belonging to separate clades (see below). In seven of the 21 species having a VP302 transcript, we recover two isoforms; in ten of the 20 species having a cnidoinsulin, we recover multiple isoforms (Table 2).

**Figure 1:**
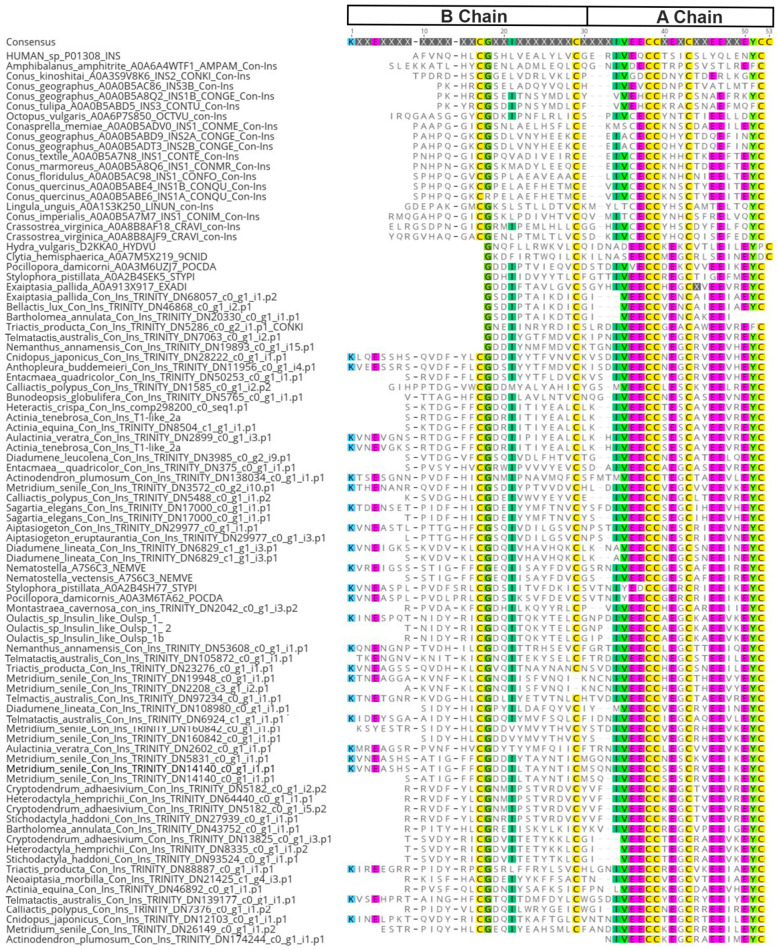
Alignment of recovered metazoan insulin-like sequences from UniProt. The alignment is comprised of the functional A and B chain for *Sea anemone* cnidoinsulins. Both chain positions are designated above. Highlighting of amino acids indicates a 75% threshold identity consensus.

Cnidoinsulin transcripts were transcribed as a singular domain transcript of 107 amino acids, with a signaling region at amino acid (AA) position 1–26 and the mature peptide at AA position 27–107. For our phylogenetic and selection analysis, we only used the functional Chain A + B, which contains six conserved cystines. From UniProt, we sourced transcripts of other metazoans known to produce insulin-like venoms and used them in our alignment (Figure 1). All the reads showed high levels of similarity across all taxa. Among *Sea anemones*, we found that chain A had more conserved AAs than chain B (Figure 1).

**Figure 2:**
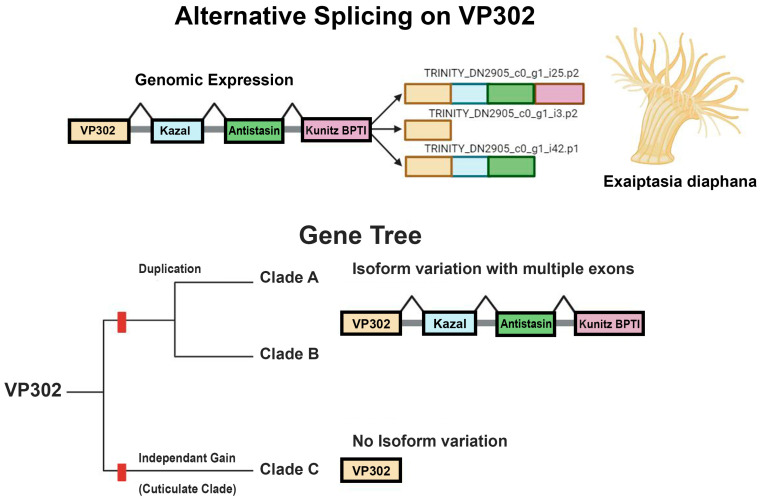
Top image shows the genomic expression of the multidomain transcript, based on the size and order of exons in transcripts from *Exaiptasia diaphana*. VP302 is followed by a Kazal domain protein > antistasin protein > Kunitz/BPTI protein. The bottom image is a schematic of the gene tree showing isoform variation across distinct clades of VP302.

**Figure 3:**
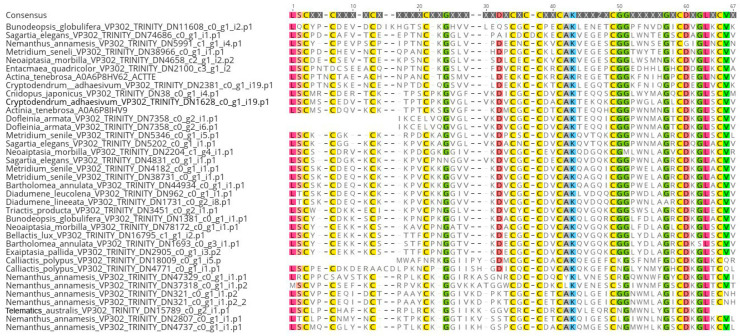
Alignment of VP302 sequences across *Sea anemones*. Highlighting of ammino acids indicates a 75% threshold identity consensus.

VP302 was found within a multi-domain transcript with 1–4 domains. The multi-domain transcript maps to a region of the genome of *Exaiptasia diaphana* (GCA_001417965.1) that is unscaffolded and annotated as a four-domain Kazal protein (Figure 2). Considering the model *Sea anemone* species *Exaiptasia diaphana* [venom302_TRINITY_DN2905_c0_g1_i25] as an example, VP302 has a signaling region at AA 1–14 and an insulin-like growth factor-binding protein (IGFBP) domain from AA 15–96. A BLAST search of this region on UniProt returned matches with IGFBP proteins from a range of taxa, but its top hit (bit score 71.1, E value 5 × 10^−13^) was to A0A8B7DI77_HYDVU, a sequence from the hydrozoan cnidarian *Hydra vulgaris* annotated as “Venom protein 302-like”. When aligned across all *Sea anemones*, VP302 was found to have a conserved region at positions 37–67 and a total of 11 conserved cystines (Figure 3).

VP302 is followed by a Kazal domain protein, encoded at AA position 100–167 in the transcript from *E. diaphana*. The top hit for this region (bit score 182, E value of 6 × 10^−52^) is to A0A913YN61_EXADI Uncharacterized protein from *E. diaphana.* Its top hit with an annotation is A0A8B6XI09_HYDVU BPTI/Kunitz domain-containing protein 4-like (bit score 179, E value 5 × 10^−10^) from *Hydra vulgaris*. An intron occurs at AA position 168–195. Following the Kazal protein, at AA 196–256, is a region that matches a PFAM domain Antistasin; the BLAST search of UniProt had as its top hit (bit score 49.1, E value 8 × 10^−5^) A0A817K5N8_9BILA, an antistasin-like protein from an unknown rotarian protist. Antistasin is better known as blood coagulation factor Xa/proclotting enzyme inhibitor and was previously noted as a possible venom constituent in clownfish-hosting anemones [9]. The region from AA 257–314 had a PFAM annotation of BPTI/Kunitz domain; its top hit (bit score 73.3, E value of 1 × 10^−14^) via a BLAST search of UniProt was to A7SDB9 A7SDB9_NEMVE, a BPTI/Kunitz inhibitor from the *Sea anemone Nematostella vectensis*. BPTI/Kunitz domain proteins are also known as venom Kunitz-type proteins.

In all sequences examined here, the four exons occurred in the same order: IGFBP (VP302) > Kazal > antistasin > Kunitz/BPTI. The studied species of *Sea anemone* show variation in number of transcripts, the length and sequence of the elements within the multidomain transcript, and the number of venom genes within those transcripts (Table 2). We found only one deviation from this canonical order: one transcript from *Actinia tenebrosa* [A0A6P8HV62_ACTTE] lacked an antistasin and had a tandem repeat of Kunitz/BPTI. All other transcripts from *Actinia tenebrosa* followed canonical order, even when they varied in the number (occurrence) of genes within the multidomain transcript.

### 2.2. Phylogenomics

**Figure 4:**
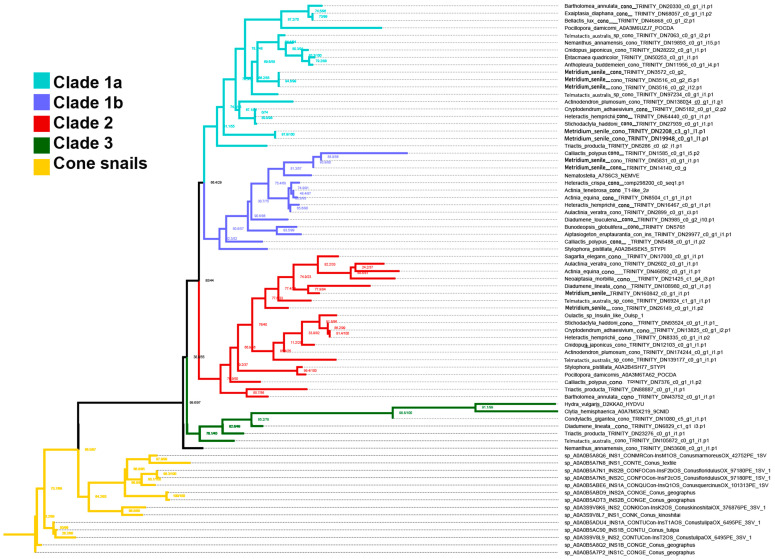
Gene tree for cnidoinsulins. This tree uses all recovered *Sea anemone* cnidoinsulins and is rooted with recovered cone snail sequences. Each clade is in a distinct color, with the key at upper right.

We find cnidoinsulin genes distributed into four clades (Figure 4). Each clade includes genes from species belonging to multiple superfamilies, but in no clade does the gene tree match the expected species tree. The earliest-branching clade, denoted Clade 3, is sister to the remaining three lineages and contains the fewest sequences, with genes from the hydrozoans *Hydra* and *Clytia* and four species of actiniarians: the metridioideans *Diadumene lineata*, *Telmatactis australis*, and *Triactis producta* and the actinioidean *Condylactis gigantea* (Figure 4). Clade 2 is the most species-rich clade, with sequences from 16 species; Clade 1A has sequences from 13 species and Clade 1B has sequences from 11 species. Clades 1 and 2 include only sequences from anthozoans, with transcripts from the scleractinians *Pocillopora damicornis* and *Stylophora pistillata* recovered in Clades 1 and 2 (Clade 1B: *S. pistillata*; Clade 2: *S. pistillata*, *P. damicornis*). In general, there are more shared species between Clade 1 and 2 than between Clades 1A and 1B: Clades 1A and 1B share only two species; Clades 1A and 2 share nine species, and Clades 1B and 2 share six species. The metridioidean *Metridium senile* and the actinioidean *Heterodactyla hemprichii* are the only species with transcripts in Clades 1A, 1B, and 2. No species has sequences in all clades, or in Clade 1, 2, and 3.

**Figure 5:**
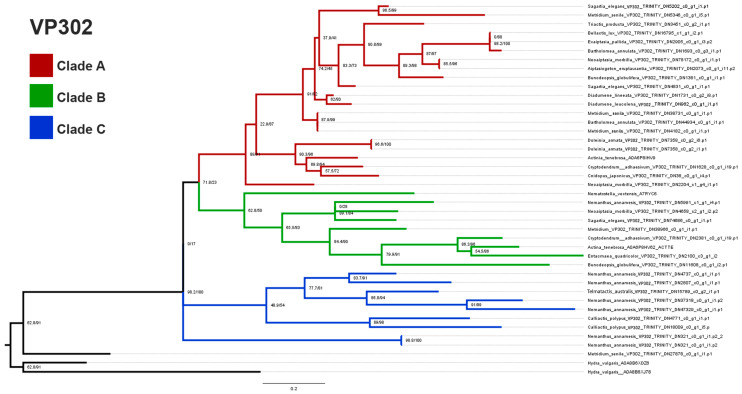
Gene tree of VP302. The tree depicts each clade in a distinct color. Clade A and B are interpreted to have arisen via a duplication event, whereas Clade C is interpreted as an independent lineage of VP302.

The gene tree from our phylogenomic analysis includes three clades of sequences for VP302 (Figure 5). Clades B and C are more closely related to each other than either is to Clade A, but precise relationships among the three clades are unclear. Clade A has the fewest number of sequences and the least diversity in terms of species, including only three species, all members of the metridioidean subclade Cuticulata: *Nemanthus annamensis, Calliactis polypus,* and *Telmatactis australis.* Clades B and C both include representatives from Actinioidea and Metridioidea and in those clades, the gene trees largely reflected relationships expected based on the species phylogeny. Clade B has sequences from nine species and Clade C has sequences from 15 species. Overlap in terms of the species that contribute sequences to each clade is greatest between Clades B and C, which share six members; Clades A and B share one species, and Clades A and C share none. Reads in Clade C only contained sequences that only coded for VP302 and not for any multidomain proteins.

### 2.3. Predicted Protein Structure

Across the four clades of cnidoinsulins, the transcripts we used to predict structure did not converge on a single structural template (Table 3). Furthermore, for no clade did we see specificity to any one template. We found that templates based on AlphaFold-predictions were used for transcripts belonging to Clade 1B from *Nematostella vectensis* and *Oulactis* sp. and for transcripts belonging to Clade 2 from *Oulactis* sp.

In general, SWISS-MODEL-predicted cnidoinsulin protein structures that did not use an AlphaFold template had low Global Model Quality Estimate (GMQE) scores (<0.50). The three SWISS-MODEL-predicted cnidoinsulin transcripts which used AlphaFold models have a GMQE score of >0.60 but <0.66. Sequence identity followed the same pattern, with non-AlphaFold sequences having a score of <0.40 and AlphaFold templates having >0.80. Coverages varied across all predicted proteins, but generally with scores > 60 (Table 3).

**Figure 6:**
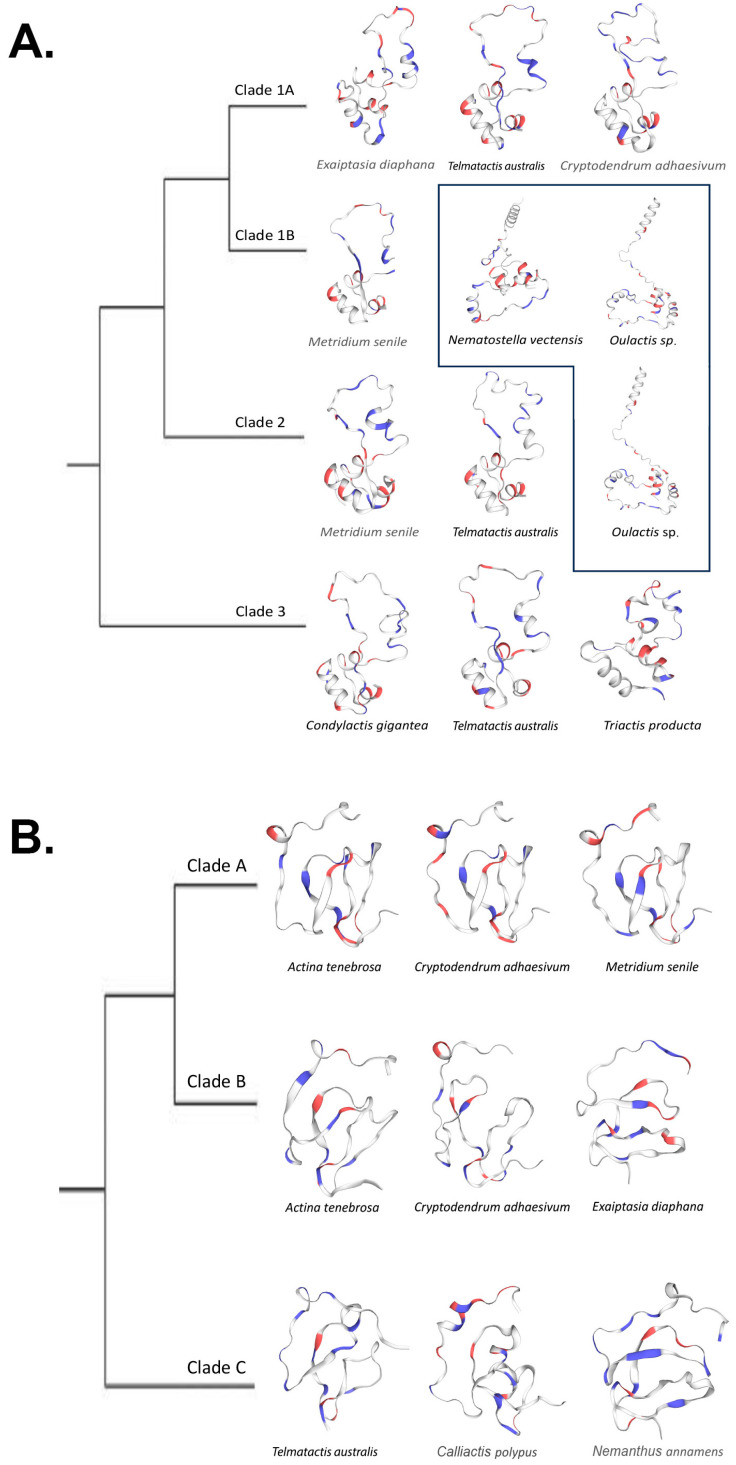
(**A**). Simplified cnidoinsulin gene tree with SWISS-MODEL-predicted models at each tip. Protein structures within a box were modeled via AlphaFold templates. (**B**). Simplified VP302 gene tree with SWISS-MODEL-predicted models at each tip.

We found that predicted protein structures for cnidoinsulins fell into two categories, those that used SWISS-MODEL templates and those that used AlphaFold templates. The predictions that used a SWISS-MODEL template all are inferred to have two canonical loops that form a large singular loop that folds back to itself and a 3–4 loop barrel. The proteins that were modeled on an AlphaFold template differed by not folding onto themselves, instead pointing away and forming a four-loop barrel (Figure 6A).

VP302 showed less variation in shape, with all but two predicted proteins matching the template [3tjq.1.A Serine protease HTRA1]. The predicted protein of *Cryptodendrum adhaesivum* from Clade B used the template [A0A6P8IHV9.1.A Four-domain proteases inhibitor-like], whereas the predicted protein for *Telmatactis australis* in Clade C matched to [1wqj.1.A Insulin-like growth factor binding protein 4]. In general, for VP302, protein structure did not vary in any significant way; it comprised two larger adjacent loops and a third incomplete loop that in some cases included a smaller tight loop (Figure 6B). All but the predicted protein for *Telmatactis australis* in clade C had a GMQE score >50. Coverage for VP302 predicted proteins was generally high (0.87–0.98) except for the predicted protein for *Actinia tenebrosa* (0.43) in Clade B (Table 3).

### 2.4. Sites under Selection

Based on the results of our gene trees for both of our venoms, we evaluated omega (dN/dS) for the alignments for each independent clade and across all clades (global). We used MEME to find sites that may have undergone episodic positive selection and the program FUBAR to infer sites under positive and negative selection. Additionally, for cnidoinsulins, we tested selection in functional chains A and B independently to determine whether the chains have distinct evolutionary pressures (Table 4). This allows us to determine whether there were clade-specific differences in the nature or strength of selection.

The global omega value (0.0211) for cnidoinsulin indicates strong negative selection as the prevailing mode of evolution. Omega ranged from 0.0075–0.156 in the constituent clades (Table 4). Despite finding no sites inferred to have undergone episodic positive selection when all sequences are considered together, when considering each clade independently, MEME detected positive selection at one site in Clade 1B, three sites in Clade 1 (Clade 1A + 1B), three sites in Clade 2, and four sites in Clade 3. Evaluating the sequences not by their clade but by their functional chain comes to a similar result: the overall value for omega is <1 for both Chain A and Chain B, with Chain A having have two sites inferred to be under positive selection and Chain B having none.

FUBAR found no sites under positive selection and 42 sites under negative selection when looking at all sequences in all clades for cnidoinsulin. No sites in Clade 1 (Clade 1A + Clade 1B), Clade 2, and Clade 3 were inferred to be under positive section, although one site within Clade 1B was inferred to be under positive selection when only those sequences were considered. FUBAR detected signatures of negative selection at 42 sites across the genes in the gene tree. Different sites were interpreted to be under negative selection in each lineage: 42 sites in Clade 1A, 32 sites in Clade 1B, 26 sites in Clade 2, and 11 sites in Clade 3. Although many of the sites are shared, the 42 sites interpreted to be under negative selection in Clade 1A and in the gene tree overall are not identical. In Clade 2, FUBAR found no sites under positive section and 26 sites under negative selection. For Clade 3, sister to both Clade 1 and 2, FUBAR found no sites under positive section and 11 sites under negative selection.

The overall omega value for VP302 was 0.172, indicating negative selection. MEME detected a total of 10 sites under positive selection when all sequences were considered. Clade A had the highest omega value (0.256) and no sites under positive selection. Clade B had the lowest omega value (0.0979), and MEME found five sites under positive selection. Clade A + Clade B had an omega value of 0.119 and seven sites inferred to be under positive selection. Clade C, sister to Clades A + B, had an omega value of 0.178 and two sites inferred to be under positive selection.

FUBAR identified no sites under positive selection when looking at all clades of sequences or when considering the sequences of each clade independently. It detected 30 sites under negative selection in Clade A, 43 sites in Clade B, and 26 in Clade C. Considering Clade B + C identified 45 sites under negative selection, including several sites not interpreted as under negative selection when the sequences of each of the constituent subclades were considered separately.

## 3. Discussion

### 3.1. Insulin-like Venoms in Sea anemones

Most species included in this study had transcripts for both cnidoinsulins and VP302. A handful of actinioidean species had only transcripts for cnidoinsulins, but only one species, *Dofleinia armata*, had transcripts for VP302 but no cnidoinsulins. The absence of transcripts for any given species may reflect the quality of the specific tissue assayed, transcriptome quality, sequencing depth, or the technology used for sequencing. Cnidoinsulins likely have tissue-specific expression [45], so transcriptomes without any cnidoinsulins may be from tissue that does not natively express insulin-like venoms. Non-expression also might reflect biological factors, such as lack of transcriptomic activity for a specific gene at the instance of sampling or population-level loss of venom [12].

### 3.2. Phylogenetics

Cnidoinsulins were the more phylogenetically widespread and commonly recovered type of ILP in the transcriptomes we studied. Contra the finding of Fu and colleagues [46], our findings indicate that cnidoinsulins have a single cnidarian-specific origin, rather than an actinarian-specific origin. Within the cnidoinsulin tree, there are multiple instances of duplication and loss (Figure 4). The gene tree for the various cnidoinsulin sequences we recovered indicates phylogenetically deep duplications in this gene, as Clades 1B, 2, and 3 include samples from across Cnidaria (Clade 3) and Anthozoa (Clade 2, 1B). Clades 1 and 2 are anthozoan-specific clades, which suggests a duplication in a common ancestor of actiniarians and scleractinians. Clade 1A appears to represent an actiniarian-specific duplication event, with the duplication either happening in the common ancestor of all Actiniaria (with loss of copies or of expression in Edwardsioidea) or in a common ancestor of Actinioidea + Metridioidea not also shared by Edwardsioidea. The phylogenetic tree for each clade of sequences did not reflect the expected species tree, suggesting loss or lower expression of cnidoinsulins in some species. Generalizing from *Sea anemones* to the other cnidarian lineages, we expect cnidoinsulins to be broadly present and to show intra-lineage gains and losses within each cnidarian lineage.

The phylogenetic evolutionary history of *Sea anemone* venom VP302 gives us a clearer picture of its evolutionary history, compared to cnidoinsulins. The topology of the tree suggests one major duplication event in VP302, leading to Clades A and B. The much more distantly related and phylogenetically restricted sequences of Clade C might represent an instance of secondary recruitment in the metridioidean subclade Cuticulata. Additionally, unlike in the cnidoinsulin tree, the topology of each clade within the VP302 tree largely reflects the expected species tree, at least at the level of superfamilies and major clades within them. We do not find VP302 sequences from other cnidarians interspersed in the phylogenetic tree of *Sea anemone* VP302 sequences.

### 3.3. Alternative Splicing

Alternative splicing is a common genetic regulatory mechanism in Eukaryotes and is commonly part of the venom systems of many species [53,54,55,56,57,58], where it has been suggested to increase the diversity of possible venoms in any one species. Alternative splicing has been identified in venom genes for *Sea anemones* only for the *Sea anemone* 8 (SA8) toxin gene family, which has alternative splice isoforms derived from an inverted SA8 gene [59].

In contrast to these examples of a single transcript that encodes multiple copies of the same venom, our inference for VP302 is that it is part of multi-domain transcript, with multiple toxins encoded on it. The transcript that includes VP302 consists of the following known venoms and their PFAM annotations: signaling region > Insulin-like Growth Factor-Binding Protein VP302 (IGFBP) > Kazal domain protein > coagulation factor Xa/proclotting enzyme inhibitor (antistasin) > serine protease inhibitor (Kunitz/BPTI). All four genes have been previously associated with venom in Cnidaria [5,7,9,49].

We recover the VP302-containing transcript with all four exons and in transcripts with one, two, or three exons. Splicing-related isoform variation occurs only in Clade A + B, suggesting that this mechanism of generating diversity in venoms is unique to that lineage of VP302 genes. Clade C, which we infer to be an independent gain of venom function for VP302 or a related IGFBP, only contained single-domain VP302 transcripts. There was variation in the number of exons in any given transcripts but not in the order in which they were encoded. We only found transcripts that differed from the 3′ end with venom coding genes being spliced 3′–5′. For each transcript we recovered that also had isoform variants, the shared exons across these isoforms were identically matched. We examined the position of these transcripts in the genome of *Exaiptasia daphnia* and found that the reads were on a nonannotated scaffold of its genome. The multi-domain transcript followed the same positional arrangement in the genome. Alternative splicing seems to be the most likely transcriptional regulation mechanism contributing to the isoform variation we captured across Clades A and B.

### 3.4. Structural Predictions

SWISS-MODEL predictions of protein structure for insulin-like venoms from select actiniarians did not show substantially different structures. The most notable variation we found in the predicted protein structure was when SWISS-MODEL used AlphaFold-derived templates for its protein prediction for cnidoinsulins. All the cnidoinsulins modeled on AlphaFold templates have a similar shape and differ from the non-AlphaFold models in not having the single loop fold onto itself (Ref to Figure 6). For both cnidoinsulins and VP302, the consistency in their predicted shape despite underlying diversity in sequence underscores the ability of venom coding genes to maintain consistent structure and, presumably, function across evolutionary divergences.

We found that most of our predicted proteins have a low GMQE score, implying a low confidence in the predicted structure. Nonetheless, the shape of the predicted structure was consistent regardless of the scores, with the most substantive difference between the models derived from AlphaFold templates and those based only on SWISS-MODEL. This could be due to the variation in the sequence itself and the shapes being consistent due to the highly conserved cysteine frameworks.

### 3.5. Selection Analysis

Our phylogenetic results demonstrate that each type of ILP inferred to be part of the venom of *Sea anemones* consists of sequences with independent histories of duplication, gain, and loss. Not only do sequences for each ILP have their own evolutionary history, but each clade of sequences has a different signature of natural selection. Cnidoinsulins as a whole (Clade (1 + 2) + 3) appear to be governed by strong negative selection, as predicted by previous work on the evolution of genes encoding venom in *Sea anemones* [14,15]. However, we found variation across cnidoinsulins in dN/dS values (Table 4). For cnidoinsulins, the clades with greater phylogenetic breadth have the weakest negative selection: omega values were highest (but below zero) for Clade 3, which included scleractinian and hydrozoan sequences in addition to sequences from actiniarians. The two chains that comprise the mature cnidoinsulin both show patterns of substitution consistent with negative selection, but negative selection is stronger in Chain A than in Chain B (Table 4).

Like cnidoinsulin, VP302 is evolving under negative selection, although the overall omega value is relatively weaker (Table 4). However, this value may be skewed by the evolutionary history of VP302, where the sequences in Clade C, the sequences more recently and phylogenetically locally recruited into venom, show weaker negative section than the VP302 sequences more prevalent across the actiniarian tree. This clear indication of distinct evolutionary trajectories within each lineage of sequences may indicate specialization of function among the various insulin-like venoms and highlighting that inferences about mode and strength of selection may be in ways that overall (global) dN/dS values simply cannot capture.

### 3.6. Sites under Selection—A Scaling Issue?

Inferences about the number of sites under selection (positive or negative) in any given alignment also change with the phylogenetic framing of the question and depend on the method of inference. Using MEME, we detected no individual AA sites in cnidoinsulin or in VP302 that had been under episodic or diversifying selection (Table 4). However, when we consider each clade within our sequence tree for cnidoinsulin or VP302, we find multiple sites under episodic or diversifying selection. Analyses using FUBAR drew the same broad picture. For cnidoinsulin, we found a single site within Clade 1B under positive selection at AA position 41. This site was not identified as under positive selection when sequences in Clade 1A and 1B were considered together as “Clade 1.” The patterns for individual sites within cnidoinsulin are highly variable across clades, with no sites inferred to be under positive selection in the sequences of Clades 1A, 2, or 3. The sequences of Clade 3 are generally the most different in terms of which sites are under selection, in both MEME and FUBAR analyses. VP302 shows a less varied and dynamic picture than cnidoinsulin, with no sites under episodic or positive selection. Unexpectedly, two sites in VP302 (AA 2, 56) are interpreted as under negative selection in each individual clade of sequences but are inferred to be neutral when lineages are aggregated into analyses of all sequences or sequences of Clade 1 + 2. Thus, we find that inferences about sites under selection is context-dependent, relative to the framing of the analysis in terms of how inclusive it is of sublineages.

**Figure 7:**
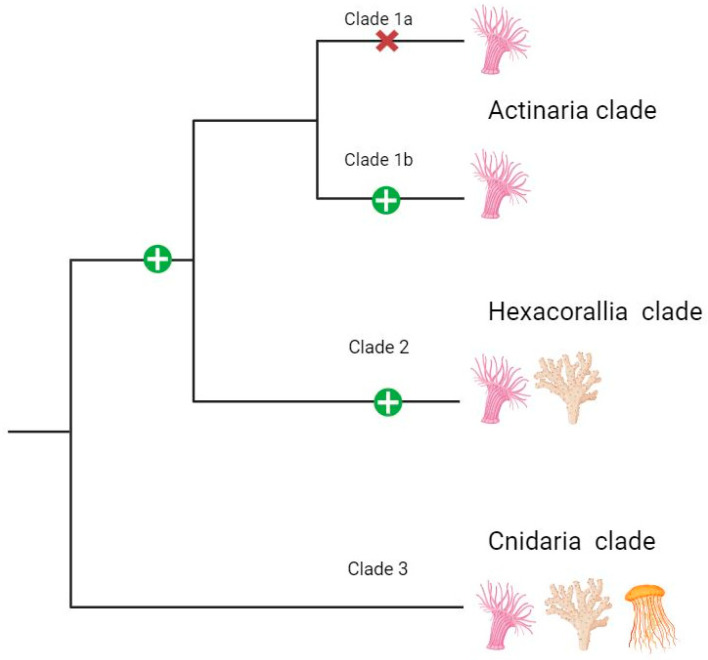
An interpretation of selection across cnidoinsulins. The taxonomic breadth of membership within each clade is indicated with the clade labels and pictograms; refer to Figure 4 for the full tree. Representation of the ancestral state of selection across the cnidoinsulin gene tree. Each clade is labeled to indicate the taxonomic breadth it encompasses. The green plus sign indicates lineages in which we found positive selection for a specific amino acid. The red “X” represents a loss in the positive selective signal for that particular amino acid.

In cases where we see sites under selection in both branches of a clade (e.g., in Clades 1A and 1B, or Clades 1 and 2), we expect that the common ancestor sequence, prior to the gene duplication (Figure 7), would have been under selection, and that the site on the protein might be one that has potential to vary (if selection is positive) or requires constraint (if selection is negative). Instances where there are differences in the evolutionary circumstances of a site between sequences in sibling clades suggest independence of evolutionary pressures on those sequences, with these different pressures reflecting different interactions in terms of the absolute function (as determined by structure) or the relative function within distinct ecological/organismal interactions. In the specific instances of cnidoinsulin and VP302, we expect that the sequences (and thus products) of Clade 3 cnidoinsulin are evolving independently of those sequences in Clades 1 and 2, and that the sequences and products of Clade C of VP302 are evolving independently and under different pressures than the sequences belonging to Clades 1 and 2. In both of these cases, the phylogenetic distribution of the sequences aligns with these predictions, as Clade 3 cnidoinsulins occur much more broadly, representing a pan-Cnidarian distribution, and as VP302 sequences of Clade C seem to represent an independent, secondary gain of sequence and function in cuticulate metridioideans.

Additionally, for cnidoinsulin, when examining both functional chains independently, Chain A has two sites inferred to be under positive selection, whereas no sites within Chain B are inferred to be under positive selection. Chain A may show a stronger signature of selection because it has more physical interactions in the molecular environments [16], and so is more directly under selection than the more internally placed chain B.

## 4. Materials and Methods

### 4.1. Annotation/Identification 

We sourced 29 transcriptomes from NCBI and collaborators using SRA Toolkit [60] and compiled unpublished transcriptomes from collaborators for two additional species, and sequenced the transcriptomes of three species, ultimately considering transcriptomes from 34 species (Supplemental Table S1). Transcriptome construction followed a Trinity v2.2 [61] de novo assembly using the Trimmomatic [62] option. Transcriptome annotation followed the pipeline from Delgado and colleagues [9], which uses Transdecoder v5.5.0 (https://transdecoder.github.io (accessed on 1 December 2023) with a changed min sequence length of 28. HMMMER [63] was used to predict protein family annotation (PFAM). From among these results, we retained for downstream analyses sequences in gene families tagged with the keyword “insulin”.

Transcripts with “insulin” PFAM annotation were annotated by querying sequences against Swiss-Prot database (9 December 2023) with BLAST [64]. We manually curated results to include only transcripts that closely matched other known venoms using an e-value cutoff of 0.001. To further confirm transcript identity, we sorted transcripts by their respective UniProt BLAST matches. For each distinct match type, we conducted a homology search for similar venom types on UniProt. Venoms have conserved cysteine frameworks [65,66,67,68], and genetic alignments are used to confirm BLAST hit annotations. We used all the sourced transcripts which matched both in PFAM annotation and homologous genes from UniProt to conduct an alignment using CLUSTAL OMEGA [69]. We visualized the resulting alignment on Geneious Prime software 2023 V11.0.18 [70]. We retained only transcripts that had matching cysteine residues.

We investigated the multi-domain transcript for VP302 by submitting all transcripts that included VP302 into InterPro 94.0 (https://www.ebi.ac.uk/interpro/ (accessed on 30 December 2023)) to identify signaling regions, protein domains, and total length of each protein. We examined the genomic positioning of VP302 in the genome of *Exaiptasia diaphana* (Baumgarten, 2015 [71]) by blasting the transcript to the reference genome [GCF_001417965.1 (Aiptasia genome 1.1)].

### 4.2. Phylogenomic Analyses

We removed signaling regions for both cnidoinsulins and VP302 prior to tree building. Because the C chain of cnidoinsulin is not part of the functional unit [35], we followed previous studies [45] and used only chains A and B for our analyses. For VP302, we isolated the transcript for that peptide from the multi-domain transcript. For both cnidoinsulin and VP302, phylogenetic trees were created using IQtree [72] on the IQtree web server (http://iqtree.cibiv.univie.ac.at/ (accessed on 3 October 2023)), which implements evolutionary model selection using Modelfinder [73].

### 4.3. Structural Predictions

We modeled the structure for three species per recovered clade in each of our gene trees. We chose individuals with possible overlap across clades, while considering phylogenetic representation of the species used. We carried out structural prediction modeling using the SWISS-MODEL workspace (https://swissmodel.expasy.org/ (accessed on 10 October 2023)) [74,75,76,77] which uses homology-based searches to find structural templates for protein modeling using the SWISS-MODEL repository [78]. All protein quality estimation was carried out within the SWISS-MODEL workspace. Proteins were viewed and annotated using Swiss-PdbViewer [79].

### 4.4. Mode of Selecetion

Assessment of the mode of evolution of each clade and the assessment of sites under pervasive and/or episodic positive selection was implemented in Mixed Effect Model of Evolution (MEME) [80]. The pervasive nature of selection was determined by using Fast Unconstrained Bayesian AppRoximation (FUBAR) [81] on the Datamonkey server [82,83,84]. All models used a 0.01 *p*-value cutoff.

## Figures and Tables

**Table 1:** Recovery table showing taxonomy of 34 species of *Sea anemones* whose transcriptomes were used in this study. Table also shows the recovery of clade-specific transcripts for cnidoinsulins and VP302.

			Cnidoinsulin				VP302		
			Clades				Clades		
Superfamily	Family	Species	1a	1b	2	3	A	B	C
Actinioidea	Actiniidae	*Actinia equina*							
		*Actinia tenebrosa*							
		*Anthopleura buddemeieri*							
		*Aulactinia veratra*							
		*Cnidopus japonicus*							
		*Condylactis gigantea*							
		*Dofleinia armata*							
		*Entacmaea quadricolor*							
		*Epiactis prolifera*							
		*Oulactis* sp.							
		*Macrodactyla doreensis*							
	Actinodendridae	*Actinodendron plumosum*							
	Heteractidae	*Heteractis crispa*							
	Stichodactylidae	*Stichodactyla haddoni*							
	Thalassianthidae	*Cryptodendrum adhaesivum*							
		*Heterodactyla hemprichi*							
Actinostoloidea	Actinostolidae	*Stomphia coccinea*							
Edwardsioidea	Edwardsiidae	*Edwardsiella carnea*							
		*Nematostella vectensis*							
Metridioidea (Cuticulate)	Andvakiidae	*Telmatactis australis*							
	Hormathiidae	*Caliactis polypus*							
	Nemanthidae	*Nemanthus annamensis*							
Metridioidea (Acuticulate)	Aiptasiidae	*Aiptasiogeton eruptaurantia*							
		*Bartholomea annulata*							
		*Bellactis lux*							
		*Exaiptasia diaphana*							
		*Neoaiptasia morbilla*							
	Aliciidae	*Lebrunia danae*							
		*Triactis producta*							
	Boloceroididae	*Bunodeopsis globulifera*							
	Diadumenidae	*Diadumene leucolena*							
		*Diadumene lineata*							
	Metridiidae	*Metridium senile*							
	Sagartiidae	*Sagartia elegans*							

**Table 2:** Variation of isoforms of VP302 transcripts, indicating the venom coding genes recovered within transcripts from each species. The clade of VP302 sequences to which the sequences recovered belong is indicated Species are organized taxonomically and across each VP302 clade based on our gene tree. The letter “x” indicates the genes which were recovered with in any one species.

			Clade			Genes Recovered within Isoforms			
Superfamily	Family	Species	A	B	C	VP302	Kazal	Antistasin	KunitzBPTI
Edwardsioidea	Edwardsiidae	*Nematostella vectensis*				x			
Actinioidea	Actiniidae	*Actinia tenebrosa*				x	x	x	x
		*Cnidopus japonicus*				x			
		*Condylactis gigantea*				x			
		*Dofleinia armata*				x			
		*Entacmaea quadricolor*				x	x		
	Thalassianthidae	*Cryptodendrum adhaesivum*				x	x	x	
	Stichodactylidae	*Stichodactyla haddoni*				x			
Metridioidea	Aiptasiidae	*Aiptasiogeton eruptaurantia*				x	x	x	x
		*Bartholomea annulata*				x	x	x	x
		*Bellactis lux*				x	x		
		*Exaiptasia diaphana*				x	x	x	x
		*Neoaiptasia morbilla*				x	x	x	x
	Aliciidae	*Triactis producta*				x	x	x	x
	Boloceroididae	*Bunodeopsis globulifera*				x	x	x	x
	Diadumenidae	*Diadumene leucolena*				x	x	x	x
		*Diadumene lineata*				x	x	x	
	Metridiidae	*Metridium senile*				x	x	x	x
	Sagartiidae	*Sagartia elegans*				x	x	x	x
	Andvakiidae	*Telmatactis australis*				x			
	Hormathiidae	*Calliactis polypus*				x			
	Nemanthidae	*Nemanthus annamensis*				x			

**Table 3:** SWISS-MODEL statistical results for both cnidoinsulins and VP302 across each clade of insulin-like venom we examined. The GMQE (Global Model Quality Estimate) shows the accuracy of the model built with the given alignment and template, normalized by the coverage of the target sequence. Sequence identity and the coverage indicate the match of the focal sequence to the template sequence.

Venom	CLADE	Species	GMQE	Template	Seq Identity %	Coverage %
Cnidoinsulin	Clade 1a	*Cryptodendrum adhaesivum*	0.3	2kqp.1.A Insulin	19	61
		*Exaiptasia diaphana*	0.37	2kqp.1.A Insulin	28	71
		*Telmatactis* sp.	0.25	3kr3.1.A Insulin-like growth factor II	35.7	47
	Clade 1b	*Metridium senile*	0.48	2kqp.1.A Insulin	27	64
		*Nematostella vectensis*	0.66	AlphaFold DB model of A7S6C3_NEMVE	100	62
		*Oulactis* sp.	0.63	AlphaFold DB model of A0A6P8J5R7_ACTTE	83.19	98
	Clade 2	*Metridium senile*	0.32	5l3m.1.A Insulin-like growth factor II	26.67	61
		*Oulactis* sp.	0.63	AlphaFold DB model of A0A6P8J5R7_ACTTE	84.96	98
		*Telmatactis* sp.	0.27	2kqp.1.A Insulin	23	32
	Clade 3	*Condylactis gigantea*	0.32	5l3m.1.A Insulin-like growth factor II	22.03	66
		*Telmatactis* sp.	0.26	7u23.1.C single-chain LCDV-1 viral insulin-like peptide	22.41	51
		*Triactis producta*	0.31	1h02.1.A INSULIN-LIKE GROWTH FACTOR I	23.33	57
VP302	Clade A	*Actina tenebrosa*	0.65	3tjq.1.A Serine protease HTRA1	45.28	91
		*Cryptodendrum adhaesivum*	0.61	3tjq.1.A Serine protease HTRA1	44.4	92
		*Metridium senile*	0.65	3tjq.1.A Serine protease HTRA1	45.28	91
	Clade B	*Actinia tenebrosa*	0.54	3tjq.1.A Serine protease HTRA1	38.64	0.43
		*Exaiptasia diaphana*	0.57	3tjq.1.A Serine protease HTRA1	43.75	0.84
		*Cryptodendrum adhaesivum*	0.83	A0A6P8IHV9.1.A Four-domain proteases inhibitor-like	75.86	0.98
	Clade C	*Calliactis polypus*	0.52	3tjq.1.A Serine protease HTRA1	38.18	0.87
		*Nemanthus annamensis*	0.51	3tjq.1.A Serine protease HTRA1	35.19	0.87
		*Telmatactis* sp.	0.43	1wqj.1.A Insulin-like growth factor binding protein 4	32.65	0.91

**Table 4:** Results of both MEME and FUBAR. MEME results give dN/dS values and the number of sites under selection. FUBAR results indicate the number of sites found under positive and negative selection.

Venom	Rate Distributions (MEME)		FUBAR	
Clade	dN/dS	Sites under (+) Selection	Sites (+)	Sites (−)
**Cnidoinsulin**				
Clade 1a	0.0125	0	0	42
Clade 1b	0.0075	1	1	32
Clade 1 (1a+1b)	0.0247	3	0	38
Clade 2	0.0557	3	0	26
Clade 3	0.156	4	0	11
All ((1+2)+3))	0.0211	0	0	36
Chain A	0.185	2	/	/
Chain B	0.212	0	/	/
**VP302**				
Clade A	0.256	0	0	30
Clade B	0.0979	5	0	43
Clade (A+B)	0.119	7	0	45
Clade C	0.178	2	0	26
All (A+B)+C)	0.172	10	0	44

## Data Availability

Not applicable.

## Supplementary Materials

The following supporting information can be downloaded at: https://www.mdpi.com/article/10.3390/md22030136/s1, Table S1: Data sources.
