# Supplementary material for: Towards the Exploration and Evolution of Insulin-like Venoms in Actiniaria (Sea anemones)
Source: Mar Drugs. 2024 Mar 20;22(3):136. doi: 10.3390/md22030136 (PMC10972341; doi:10.3390/md22030136)
Supplement: Supplementary file 1 [file marinedrugs-22-00136-s001.zip › marinedrugs-2874994-supplementary.pdf]

Superfamily	Family	Species	DATA
Actinioidea	Actiniidae	*Actinia equina*	Genome
		Actinia tenebrosa	SRR3210696
		Anthopleura buddemeieri	SRR3193961
		Aulactinia veratra	SRR3205708
		Cnidopus japonicus	SRR2134407
		Condylactis gigantea	ERR2045165
		Dofleinia armata	ERR6139597
		Entacmaea quadricolor	Ref. Delgado 2022
		Epiactis prolifera	Collborators
		Oulactis sp.	ERR2710216
		Macrodactyla doreensis	Ref. Delgado 2022
	Actinodendridae	*Actinodendron plumosum*	SRR10500343
	Heteractidae	*Heteractis crispa*	Ref. Delgado 2022
	Stichodactylidae	*Stichodactyla haddoni*	Ref. Delgado 2022
	Thalassianthidae	*Cryptodendrum adhaesivum*	Ref. Delgado 2022
		Heterodactyla hemprichi	Ref. Delgado 2022
Actinostoloidea	Actinostolidae	*Stomphia coccinea*	Collborators
Edwardsioidea	Edwardsiidae	*Edwardsiella carnea*	SRR6480800
		Nematostella vectensis	Genome
Metridioidea (Cuticulate)	Andvakiidae	*Telmatactis australis*	SRR3225580
	Hormathiidae	*Caliactis polypus*	RR3205762
	Nemanthidae	*Nemanthus annamensis*	SRR3228732
Metridioidea (Acuticulate)	Aiptasiidae	*Aiptasiogeton eruptaurantia*	Sequenced
		Bartholomea annulata	Collborators
		Bellactis lux	Sequenced
		Exaiptasia diaphana	SRR13526542
		Neoaiptasia morbilla	Sequenced
	Aliciidae	*Lebrunia danae*	Collborators
		Triactis producta	SRR8297742
	Boloceroididae	*Bunodeopsis globulifera*	Collborators
	Diadumenidae	*Diadumene leucolena*	Collborators
		Diadumene lineata	SRR8297741
	Metridiidae	*Metridium senile*	SRR6480802
	Sagartiidae	*Sagartia elegans*	Collborators

Supplemental Table S1. Samples name and origin of data. Samples either were retrived from NCBI using SRAs, gatherted from collaborators and or sequenced for this study.
